# Stages of change, treatment outcome and therapeutic alliance in adult inpatients with chronic anorexia nervosa

**DOI:** 10.1186/1471-244X-13-111

**Published:** 2013-04-09

**Authors:** Johannes Mander, Martin Teufel, Katharina Keifenheim, Stephan Zipfel, Katrin Elisabeth Giel

**Affiliations:** 1 Department of Psychosomatic Medicine and Psychotherapy, Medical University Hospital Tübingen, Osianderstr. 5, 72076 Tübingen, Germany

**Keywords:** Alliance, Anorexia nervosa, Chronic, Eating disorder, Inpatient, Motivation, Therapy, Stages of change, Transtheoretical model

## Abstract

**Background:**

Anorexia nervosa (AN) is associated with high rates of chronicity and relapse risk is a considerable therapeutic challenge in the disorder. The aim of the present study was to investigate the association of stages of change and outcome with a focus on the relapse struggle in the maintenance stage in patients with predominantly chronic AN. Further, therapeutic alliance and stages of change associations were explored.

**Methods:**

As an instrument measuring relapse struggle in the maintenance stage, we applied the short form of the University of Rhode Island Change Assessment-Short (URICA-S). We assessed stages of change in 39 patients with a predominantly chronic course of AN in early, middle, and late stages of inpatient psychotherapy. General symptom severity as assessed by the SCL-90-R and weight change were investigated as outcome measures.

**Results:**

In-line with earlier evidence, *contemplation* significantly predicted therapeutic alliance. Further, we demonstrated that relapse risk as operationalized by URICA-S *maintenance* is an important predictor of general psychopathology. BMI change was not predicted by stages of change.

**Conclusions:**

The URICA-S *maintenance* scale might be applied to help identify patients at relapse risk. High URICA-S maintenance scores could be considered as one critical aspect of AN patients who might especially benefit from relapse-preventing aftercare programs.

## Background

Anorexia nervosa (AN) has been recognized as a severe disorder that is challenging to treat. This is partly reflected by the often long and severe course of the disorder, which in many patients is characterized by recurrent treatment attempts and dropouts, rehospitalization, high rates of relapses and chronicity [1-3]. The comparably large group of patients with long-lasting problems and a chronic course has been identified as being particularly difficult to treat and having poor treatment outcomes [4]. Generally, evidence on treatment approaches for long-standing AN is very limited [4] and more knowledge on predictors of treatment response and resistance in AN patients could inform the further development of such approaches. Therefore, the aim of the present study was to investigate the association of the motivational stages of the patient with treatment outcome variables and therapeutic alliance in chronic AN patients.

One major challenge that has been identified in AN treatment is the ambivalent motivation for change and treatment in affected patients. This ambivalence has been associated with the pronounced egosyntonicity of AN symptoms, including severe underweight, as a remarkable feature of the disorder, which constitutes a major barrier to behavioral change [5]. Two approaches targeting motivation in therapeutic change are the Transtheoretical Model (TTM) by Prochaska & DiClemente [6,7], as a more theoretical framework, and Motivational Interviewing [8], as a treatment approach specifically targeting the enhancement of motivation. Both approaches have recently been adopted in the field of eating disorders, including AN [9]. The TTM suggests that therapeutic interventions should be adapted to the motivational stage of the patient. It distinguishes four empirically validated stages of change: In the *precontemplation* stage, patients have no intention for a therapeutic change. In the *contemplation* stage, patients ambivalently think about therapeutic change. In the *action* stage, active work on therapeutic change is central and in the *maintenance* stage, patients focus on relapse prevention. One central assumption of the TTM is that patients move through the stages of change in a spiral pattern. Hence, relapses are regarded as integral parts in the change cycle [10]. The most widely used dimensional instrument to measure the stages of change is the University of Rhode Island Change Assessment (URICA, [11]). Recently, a short version with excellent psychometric properties has been constructed [12]. A meta-analysis found robust effect size of d = .46 for the association between URICA stages of change and outcome [13]. The URICA comprises several items that clearly reflect relapse struggle [12,14]. Hence, it is an instrument that specifically identifies patients at relapse risk. This is of importance concerning AN research, because, as we have outlined above, high rates of relapses and chronicity are critical elements in AN treatment [15].

In their recent review, Dray & Wade [9] point out that while there is first evidence that the TTM is applicable to eating disorders and the stages of change are associated with treatment outcome in affected patients, only few studies have been conducted and more research is needed. The investigation of different patient samples (e.g. age groups, patients with a chronic course), a broader focus on different relevant treatment outcomes, and the consideration of the therapeutic alliance have been identified as major research gaps [9]. To our knowledge, there are currently only three studies published on the predictive value of stage of change on treatment outcome in AN patients [16-18], two of which were conducted in adolescent samples. All of them report on a global score of stages of change as an indicator of motivation to change and identified this global score as a positive predictor of weight gain, improvement of eating disorder pathology, and composite improvement outcome at discharge [17,18] as well as of weight maintenance after discharge [16].

In the present study, we have investigated the predictive value of stages of change in an adult sample of patients receiving inpatient treatment for AN. As outlined above, relapses and chronicity are major concerns in AN treatment. Hence, we investigated adult patients who suffered at least one year from full syndromal AN, and predominantly focused on chronic patients. Complementing earlier evidence and addressing current research gaps outlined by the recent review by Dray & Wade [9], we have focused on different relevant outcomes in AN treatment, including weight gain, general psychopathology, and therapeutic alliance. In-line with earlier evidence on the role of stages of change in the therapeutic process of inpatient psychotherapy by our work group [12], we hypothesized that lower ratings on precontemplation as well as higher ratings on contemplation and action are associated with a better outcome in adult AN inpatients in terms of weight gain and improvement of general psychopathology. Further, we presumed that higher ratings on the maintenance scale are associated with a more negative outcome, because the maintenance score of the URICA-S reflects the struggle with relapses [12]. Turning to the therapeutic alliance, we hypothesized in-line with earlier evidence [19] that contemplation at an earlier stage of therapy is positively associated with the alliance at a later stage of therapy. We further hypothesized that precontemplation and maintenance at earlier stages of therapy would be negatively associated while action would be positively associated with therapeutic alliance at later stages of therapy.

## Method

### Measures

All patients were medically assessed with the Structured Clinical Interview for DSM-IV, German version (SCID-I, [20]). The German version of the Symptom-Checklist-90-Revised (SCL-90-R, [21]) was administered to all patients as a measure of general symptom severity. The German version of the Symptom-Checklist-90-Revised (SCL-90-R, [21]) is a measure of general symptom severity. It consists of eleven subscales, with 90 items on a 5-stepped scale. It showed excellent internal consistencies, with .79 ≤ α ≤ .89 and good retest-reliabilities, with .69 ≤ *r* ≤ .92, and acceptable construct validity, with scale-outcome correlations between .27 ≤ *r* ≤ .81.

Further, each patient filled in the German short form of the URICA (URICA-S [12]). The original long form of the URICA consists of 32 items with 8 items for each of the 4 subscales *precontemplation*, *contemplation*, *action,* and *maintenance*. The URICA-S consists of 16 items rated on a scale from 0 (very untrue) to 4 (very true) and measures four stages of change (*precontemplation*, *contemplation*, *action*, *maintenance*) with 4 items per subscale. In a sample of 253 patients, Mander et al. (12) demonstrated an excellent factor structure with factor loadings of .52 ≤ λ ≤ .87. Confirmatory factor analyses supported the exploratory model. The instrument revealed acceptable to excellent internal consistencies, with .61 ≤ α ≤ .84. The measure demonstrated high correlations to the subscales of the original long form of the URICA, with .83 ≤ *r* ≤ .96. Referring to the criterion-related validity, outcome was significantly predicted by the *contemplation*, *action,* and *maintenance* subscales as well as by the *committed action* global score. As the *maintenance* score URICA-S refers to the struggle with relapses, it might be especially important to identify patients at risk for further chronicity. Therefore, more negative values in *maintenance* reflect more problems with relapses.

Each patient completed the Scale for the Multiperspective Assessment of General Change Mechanisms in Psychotherapy (SACiP) [22]. It is a measure with the six dimensions *resource activation*, *problem actuation*, *mastery*, *clarification of meaning*, *emotional bond*, with three items each and *agreement on collaboration*, which comprises the aspects *tasks* and *goals*, with six items. The two dimensions *emotional bond* and *agreement on collaboration* refer to Bordin´s [23] concept of the therapeutic alliance. The SACiP consists of 21 items, which are rated on a 5-stepped scale ranging from 0 (not correct at all) to 4 (fully correct) with correspondingly formulated items from patient and therapist perspective. The measure demonstrated an excellent factor structure with factor loadings of .51 ≤ λ ≤ .85. Confirmatory factor analyses supported the exploratory model. The instrument revealed good to excellent internal consistencies, with .71 ≤ α ≤ .90. Referring to the criterion-related validity, outcome was significantly predicted by all change mechanisms except for *problem actuation*.

### Treatment and study design

Specific inclusion criteria were a SCID-I primary diagnosis of full-syndrome AN. General exclusion criteria were as follows: (1) Duration of disorder of less than one year, (2) an age below 18 or above 59 years, (3) insufficient German language skills, (4) psychotic or substance-related disorders. All patients completed an inpatient treatment at the Department of Psychosomatic Medicine and Psychotherapy of Tübingen University, Germany. The treatment consisted of individual therapy, group therapy, art therapy, and music therapy two times a week. Psychotherapy comprised a cognitive-behavioral therapy with supplementary interpersonal psychotherapeutic elements. Eight (7 female) psychotherapists with at least one year of experience conducted therapy. Individual therapy was conducted two times a week. Duration of inpatient treatment comprised a minimum of 16 and a maximum of 84 days, with a mean of 48.8 (SD 16.4). Median of treatment was 48.5 days. Patients below the median had a mean treatment duration of 33.9 (SD 8.6) days and patients above the median had a mean treatment duration of 60.7 (SD 9.7) days. Patients were assessed at baseline (t_1_), after the 7th session (t_2_) and after the last session (t_3_), respectively. The patients with a shorter treatment duration than 30 days were assessed only at 2 measuring times. The initial SCID-I assessment was conducted by three PhD-students who completed a university-based training. A university-affiliated expert regularly supervised them. The local ethics committee of the medical faculty of the university of Tübingen approved the study protocol (project number 29/2009B02) in accordance with the Helsinki Declaration. All participants provided written informed consent prior to study participation.

### Statistical analyses

To identify empirical associations between stages of change and outcome as well as therapeutic alliance, we first of all calculated a set of correlational analyses. Further, concerning general psychopathology, we calculated three hierarchical regression analyses where we blockwise entered the four URICA-S subscale scores as well as the autoregressor (SCL-90 global severity index at t_1_), one for each measuring time. As the dependent variable, we always used the SCL-90 global severity index at t_3_. In the first block, we entered the autoregressor of the SCL-90. In the second block, we always entered the maintenance score because it demonstrated the highest association to therapy outcome in the correlational analysis. The order in which the other subscales were entered in the following blocks depended on the strengths of their correlations with therapy outcome. In a similar way, we implemented predictive models for BMI at t_3_ and therapeutic alliance at t_3_ as the dependent variables.

## Results

### Clinical and socio-demographic sample description

Thirty-nine inpatients treated for AN participated in the study. Complete data sets were available for 35 patients and for 28 at the end of treatment. The demographic data did not significantly differ between the full sample and the sample at the end of treatment. A total of 97.1% of patients were female. On average, patients were 27.7 (SD 10.63) years of age; 23.1% were married, 41.0% lived with parents, 35.9% had an A-level degree, 38.5% had a formal professional qualification, 20.5% were employed, 35.9% were still in job training and 20.4% were unemployed. The percentage of patients with a diagnosis of a major depression as comorbidity was 56.4%. Average BMI at therapy entrance was 14.94 kg/m^2^ (SD 1.96), with a minimum of 11.50 and a maximum of 17.40. Average BMI at end of therapy was 16.19 (SD 2.04) with a minimum of 12.00 and a maximum of 20.16. Mean duration of full syndromal AN was 8.6 years (SD 8.84), with a minimum of 1 and a maximum of 27 years and a median of 3.0 years. The percentage of those having a 1 to 3 year duration of the disorder was 45.7%, while 22.9% had a duration of the disorder between 3 and 6 years and 31.4% had a duration of the disorder of more than 6 years.

### Stages of change association to treatment outcome

Correlational analyses revealed that the association of *maintenance* with the SCL-90 was highly significant, but there was no correlation of *maintenance* with the BMI. *Precontemplation* was neither associated with the SCL-90 nor with the BMI. Association of *contemplation* at t_2_ with the SCL-90 was tentatively positive and with the BMI at t_3_ was significantly positive. Association of *action* at t_2_ and t_3_ was tentatively negative with the SCL-90 and positive with the BMI. Concerning stages of change and therapeutic alliance associations, correlational analyses revealed, as predicted, that association of *precontemplation* at t_1_ was significantly negative and *contemplation* at t_1_ was significantly positive with *emotional bond* and *agreement on collaboration*. For action and maintenance, no significant effects were observable. Correlational coefficients are depicted in Table 1.

**Table 1:** Correlations of the URICA subscales at all three measuring times with SCL, BMI, and therapeutic alliance at the end of therapy

	**Precontemplation**	**Contemplation**	**Action**	**Maintenance**
SCL-90				
t_1_	.09	.08	−.05	.51**
t_2_	−.11	.34	−.37	.56**
t_3_	.16	−.03	−.31	.55**
BMI				
t_1_	−.02	.01	.17	−.22
t_2_	−.13	.39	.19	.28
t_3_	.07	.24	.33	−.28
BMI change t_1_ - t_3_				
t_1_	−.07	−.07	−.08	.02
t_2_	.40*	−.12	.04	.06
t_3_	.15	−.07	−.14	−.18
Emotional bond				
t_1_	−.51*	.51*	.08	.02
t_2_	.21	.19	.03	.23
t_3_	.32	.10	.08	.14
Agreement on collaboration				
t_1_	−.50*	.46*	.29	.19
t_2_	−.38	.24	.11	.23
t_3_	−.03	.30	−.01	.14

t_1_/t_2_/t_3_ = at baseline/7^th^/last therapy session; ^*^ = *p* < .05; ^**^ = *p* < .01; ^***^ = *p* < .001.

### Stages of change as predictors of treatment outcome and therapeutic alliance

For all three measuring times, only the *maintenance* scale proved to be a significant predictor of outcome. The *maintenance* score was a significant predictor of outcome at t_1_, *R*^2^ = .60, *p* < .001; at t_2_, *R*^2^ = .62, *p* < .001; and t_3_, *R*^2^ = .53, *p* < .001. None of the other stages of change proved to be a significant predictor in any regression model. We found no significant predictive effect on BMI for any of the stages of change. Concerning predictive effects on therapeutic alliance, the contemplation score significantly predicted emotional bond, *R*^2^ = .45, *p* < .01; as well as agreement on collaboration, *R*^2^ = .40, *p* < .01. None of the other stages of change proved to be a significant predictor in any regression model. For parsimonious reasons, we report only the significant regression models concerning therapeutic outcome in Table 2 and concerning therapeutic alliance in Table 3.

**Table 2:** Significant linear regression coefficients of stages of change predicting SCL-90 symptomatology at the end of therapy (N=26)

						**95% CI**		
	**B**	**SE B**	**β**	**t**	**p**	**Lower bound**	**Upper bound**	**R**^**2**^
Model 1: Maintenance t_1_								.60
Constant	−.14	.20		−.70	.490	−.55	.27	
SCL-90 baseline	.67	.15	.61	4.38	<.001	.35	.98	
Maintenance t_1_	.17	.07	.34	2.43	.023	.03	.31	
Model 2: Maintenance t_2_								.62
Constant	−.19	.21		−.90	.38	−.64	.25	
SCL-90 baseline	.65	.16	.58	4.03	.001	.31	.98	
Maintenance t_2_	.20	.08	.38	2.59	.017	.04	.36	
Model 3: Maintenance t_3_								.53
Constant	−.08	.26		−.31	.760	−.62	.46	
SCL-90 baseline	.58	.20	.52	.29	.010	.16	1.0	
Maintenance t_3_	.16	.09	.34	1.9	.08	−.20	.35	

t_1_/t_3_ = at baseline/end of therapy. Model 1 / Model 2 / Model 3 = significant stages of change predictors at baseline/7^th^/last therapy session for SCL-90 at the end of therapy.

**Table 3:** Significant linear regression coefficients of stages of change at therapy entrance predicting therapeutic alliance at the end of therapy (N=22)

						**95% CI**		
	**B**	**SE B**	**β**	**t**	**p**	**Lower bound**	**Upper bound**	**R**^**2**^
Model 1: Emotional bond at t_3_								.45
Constant	.71	.62		1.13	.272	−.60	2.81	
Emotional bond t_1_	.40	.16	.44	2.54	.020	.07	.73	
Contemplation t_1_	.41	.17	.42	2.44	.025	.06	.76	
Model 2: Agreement on collaboration at t_3_								.40
Constant	.35	−67		.52	.61	−1.05	1.75	
Agreement on Collaboration t_1_	.47	.19	.44	2.42	.026	.06	.87	
Contemplation t_1_	.41	.19	.38	2.12	.047	.01	.81	

t_1_ = at baseline. Model 1 = stages of change at baseline significantly predicting emotional bond at the end of therapy. Model 2 = stages of change at baseline significantly predicting agreement on collaboration at the end of therapy.

## Discussion

The aim of the present study was to investigate the association of stages of change according to the TTM [6,7] with different treatment outcome variables in a sample of predominantly chronic full syndromal AN patients. Addressing current research gaps outlined by a recent review [9], we investigated the predictive value of stages of change for weight gain as assessed by changes in BMI, general psychopathology as assessed by the SCL-90, and therapeutic alliance as assessed by the SACiP [22]. As we investigated a mainly chronic sample and because high relapse rates are known to be one of the core problems of AN treatment, we applied the URICA-S as a measure of stages of change because its *maintenance* scale reflects relapse threat [12,14]. (See Appendix for detailed wording of the items of the maintenance scale).

To our knowledge, our study is the first analyzing outcome profiles in AN patients for discrete stages of change. The few earlier studies available predicted outcome by applying a global score [16-18]. We were especially interested in therapy processes in patients treated for chronic AN, and our sample therefore shows a high average duration of illness of about 9 years.

We identified significant predictive effects of the *maintenance* scale on general psychopathology for all three measuring times. The higher the *maintenance* score, that is, the higher the struggle of patients with relapses, the higher general psychopathology in the SCL-90. No other stages-of-change scales resulted in additional predictive effects on therapeutic outcome in the regression models. Hence, the *maintenance* score of the URICA-S as an indicator of relapse threat obviously is especially important to identify AN patients who improve during inpatient treatment and those who suffer from repeated drawbacks. In many patients, the course of AN is characterized by recurrent relapses, and the first time after hospital discharge seems to be a specifically vulnerable phase [15,24]. It might therefore be speculated that those patients who score lower on *maintenance*, that is, who struggle less with relapses, have taken a perspective on their disorder that understands recovery as a process incorporating relapses, and have developed strategies to cope with drawbacks and both could facilitate the success of inpatient treatment. This, in turn, would first underline the importance of integrating elements of relapse prevention into inpatient treatment programs for AN patients as, e.g. education and skills training related to drawbacks and relapses, and would second underline the importance of relapse prevention interventions following inpatient treatment. Both approaches might improve the course of the disorder, particularly in patients with long-standing problems, as Long, Fitzgerald and Hollin [25] recently reported in a prospective follow-up study that those chronic AN patients had a better outcome who received continuity of care, that is, who had outpatient treatment immediately following inpatient care. The URICA *maintenance* scale might be applied as one aspect of a relapse identification tool, which could be used to identify subgroups of AN patients who might especially benefit from such relapse-addressing aftercare programs [9,26].

In our study, stages of change surprisingly had no predictive effect on BMI. This is in contrast to an earlier study where Castro-Fornieles et al. [16] identified stages of change as a highly significant predictor of BMI at discharge. Possibly, this may be attributed to the different measures applied to define stages of change and different samples under investigation. While we are reporting data of adult patients with long-lasting full syndromal AN, Castro-Fornieles et al. [16] investigated an adolescent sample. According to state-of-the- art practice in adolescent therapy, patients were treated to normal-weight status. Further, as we investigated patients who mostly suffered for many years from AN symptoms, smaller weight change has to be expected than in adolescent patients whose eating disorder just emerged. Hence, the pre-post change in BMI was much larger in this adolescent sample (15.5 to 18.4) than in our predominantly chronic adult sample (14.9 to 16.2), which received on average much shorter inpatient treatment. Hence, more potential variance remains to be explained in weight gain in the study by Castro-Fornieles et al., which probably is responsible for the better predictive value of stages of change on BMI. However, it is generally difficult to evaluate our finding on weight gain as evidence on stages of change in adult samples is scarce [9]. Further research is needed to investigate the predictive value of stages of change on weight gain as a pivotal aim and outcome of AN treatment.

Turning to the predictive effect of stages of change at therapy entrance on therapeutic alliance on discharge, we identified significant predictive effects of the *contemplation* scale on both *emotional bond* and *agreement on collaboration* at discharge. This is in line with earlier evidence [19], where contemplation at baseline predicted positive therapeutic alliance at session one and session three in a sample of sixty patients with mixed psychiatric diagnoses. Although *precontemplation* at therapy entrance did correlate significantly and negatively with emotional bond and agreement on collaboration at discharge, it was not a significant predictor in the regression analyses when controlled for the autoregressor. AN patients often value many aspects of their disorder [5] and therefore are at least in parts or periodically characterized by *precontemplation* with regard to therapeutic change. This constitutes a major difficulty in AN treatment and assigns therapists with the motivational task to challenge *precontemplation* and try to induce *contemplation* in patients. Techniques of Motivational Interviewing [8] have been specifically designed to address this aspect and to reach patients in between *precontemplation* and *contemplation*. Our data shows that *contemplation* is a predictor of a positive therapeutic alliance in the course of inpatient treatment. This might lead to the conclusion that early intervention strategies to induce *contemplation* in AN inpatients are not only important for therapeutic change, but might also form a basis for a stable therapeutic alliance that even allows for the common cautious exploration of further stages of change in the course of treatment [8].

Our study implied several limitations as discussed hereafter. First, the URICA is sensitive to identify relapses, but it is not a disorder-specific instrument. Hence, in future studies it might be important to address patients’ relapse threats with a disorder-specific measure. The Anorexia Nervosa Stages of Change Questionnaire (ANSOCQ) [27] is an instrument measuring stages of change specifically in AN patients. In contrast to the URICA, where the *maintenance* score reflects relapse struggle, in the ANSOCQ, this score refers to the ability to maintain progress [12,14]. Nevertheless, as we have outlined above, relapse measures are of importance concerning AN research, because high rates of relapses are critical elements in AN treatment [15]. Therefore, it might be of interest to phrase a new, more differentiated disorder-specific stage-of-change scale, containing a progress maintenance as well as a relapse subscale. Second, our study predominantly included chronic inpatients. Effects of stages of change on outpatients and non-chronic patients might be different. Third, we only applied a measure of general psychopathology as a symptomatic outcome instrument. Future studies should investigate stages of change outcome associations with measures that are more specific to the eating disorder symptoms, which are at the core of AN. Fourth, the duration of inpatient treatment varied across participants. Treatment duration might influence the quality of therapeutic alliance. More specifically, in our sample, patients with longer versus shorter treatment duration expressed slightly higher therapeutic alliance ratings. As sample size in our study was too small to analyze further differences between these two groups, future research should address this aspect in studies with larger sample sizes.

## Conclusion

To sum up, our study was the first to investigate different motivational profiles on discrete stages of change as well as associations of stages of change and therapeutic alliance in adult inpatients with a long-lasting history of AN. We demonstrated that relapse risk as operationalized by the *maintenance* scale of the URICA-S is an important predictor of general psychopathology over the course of inpatient treatment in AN patients. In line with earlier evidence, *contemplation* significantly predicted therapeutic alliance. The URICA-S *maintenance* scale might be applied as a relapse identification tool. Future research should address this aspect in randomized clinical trials.

## Appendix

### Items of the URICA-S maintenance scale

It worries me that I might slip back on a problem I have already changed, so I am here to seek help. / I'm not following through with what I had already changed as well as I hoped, and I'm here to prevent a relapse of the problem. / I'm here to prevent myself from having a relapse of my problem. / It is frustrating, but I feel I might be having a recurrence of a problem I thought I had resolved.

## Competing interests

None of the authors declares financial or non-financial competing interests related to the present study.

## Authors’ contributions

JM, MT and SZ have contributed to conception and design of the study, JM and KK have performed acquisition and analysis of data, JM and KEG have contributed to interpretation of data and drafted the manuscript, MT, KK and SZ have critically revised the manuscript for important intellectual content. All authors have approved the final version of the manuscript.

## Pre-publication history

The pre-publication history for this paper can be accessed here:

http://www.biomedcentral.com/1471-244X/13/111/prepub
